# Micro-mechanical blood clot testing using smartphones

**DOI:** 10.1038/s41467-022-28499-y

**Published:** 2022-02-11

**Authors:** Justin Chan, Kelly Michaelsen, Joanne K. Estergreen, Daniel E. Sabath, Shyamnath Gollakota

**Affiliations:** 1 Paul G. Allen School of Computer Science and Engineering, University of Washington, Seattle, WA USA; 2 Department of Anesthesiology & Pain Medicine, University of Washington, Seattle, WA USA; 3 Department of Laboratory Medicine & Pathology, University of Washington, Seattle, WA USA

**Keywords:** Assay systems, Translational research, Biomedical engineering

## Abstract

Frequent prothrombin time (PT) and international normalized ratio (INR) testing is critical for millions of people on lifelong anticoagulation with warfarin. Currently, testing is performed in hospital laboratories or with expensive point-of-care devices limiting the ability to test frequently and affordably. We report a proof-of-concept PT/INR testing system that uses the vibration motor and camera on smartphones to track micro-mechanical movements of a copper particle. The smartphone system computed the PT/INR with inter-class correlation coefficients of 0.963 and 0.966, compared to a clinical-grade coagulation analyzer for 140 plasma samples and demonstrated similar results for 80 whole blood samples using a single drop of blood (10 μl). When tested with 79 blood samples with coagulopathic conditions, the smartphone system demonstrated a correlation of 0.974 for both PT/INR. Given the ubiquity of smartphones in the global setting, this proof-of-concept technology may provide affordable and effective PT and INR testing in low-resource environments.

## Abstract (web summary)

Therapy with anticoagulants requires frequent monitoring. Here the authors describe a proof-of-concept study of a simple and affordable blood clot test that uses a smartphone’s vibration motor and camera to track micro-movements in a single drop of blood.

## Introduction

The human body responds to injury with bleeding, followed by clot formation and eventually lysis^1^. This carefully maintained homeostasis minimizes the risks of hemorrhage and inappropriate clotting like ischemic stroke, myocardial infarction or pulmonary embolus^2^. However, for millions of people, medical conditions such as atrial fibrillation, mechanical heart valves and genetic mutations increase the risk of morbidity and mortality from blood clotting^2^. These individuals require lifelong administration of anticoagulation drugs such as warfarin, an effective medication but also one of the most common causes of hospitalization due to adverse drug events^3^. Hence, medication effects must be closely monitored via frequent prothrombin time (PT) or international normalized ratio (INR) tests to assess coagulation properties due to the drug’s narrow therapeutic index and interactions with food and other medications^4^. While newer anticoagulants that do not rely on regular PT/INR testing are increasing in popularity, studies show that warfarin remains the most commonly prescribed outpatient blood thinner^5^.

PT/INR testing monitors extrinsic and common pathways of the coagulation cascade. These tests are usually performed in a laboratory on expensive equipment after separating plasma from whole blood. Home PT/INR monitors directly utilize blood and have been shown to lengthen the amount of time spent in the therapeutic range, decrease the risk of thromboembolism in young patients and improve patient satisfaction and quality of life^6,7^. Time spent in the therapeutic window benefits patients with non-valvular atrial fibrillation on warfarin, since the risk of bleeding is five times higher with overly aggressive anticoagulation and the risk of ischemic events is three times higher with insufficient anticoagulation compared to levels in the therapeutic range^8^.

Despite the existence of several home PT/INR testing modules, access to affordable and accurate PT/INR testing remains a challenge. Patients in the United States are in the therapeutic range only about 64% of the time^6,9^. Patients in developing countries like Botswana, Uganda, and India are in this range, only 40% of the time due to less frequent testing^9–11^. Despite the potential for improvements from home PT/INR testing^9^, these devices cost hundreds of dollars, limiting their utility in resource-constrained environments^12,13^.

Here, we describe a proof-of-concept system that uses the vibration motor and camera on existing smartphones to perform PT/INR testing. Smartphones are increasingly becoming ubiquitous in resource-constrained environments and developing countries both in rural and urban settings^14–16^. Vibration motors and cameras have been an integral part of smartphones for more than a decade. Repurposing these smartphone sensors for PT/INR testing could enable a more affordable blood clot testing tool.

Our system visually tracks the micro-mechanical movements of a small copper particle in a cup with either a single drop of whole blood or plasma and the addition of activators. No additional electronic components are required beyond a lightweight plastic attachment that couples the phone’s vibrations to the cup. When the mixture is in a fluid state, the copper particle moves freely with the phone’s vibration. As the blood clots, the viscous mixture causes the particle to slow to a stationary state. Using computationally efficient video analytic algorithms that run on the smartphone in under 37 ms, we analyze the particle’s motion in under one minute to determine the PT/INR values. Our system can run on older smartphones such as second-hand iPhone 5s phones that were released in 2013 and cost $35. Making coagulation testing accessible in this manner may help improve time within the therapeutic range for anti-coagulation users, particularly in rural locations.

## Results

### Concept and prototype

Our design builds on conventional central laboratory and point-of-care coagulation tests. Since manual visual detection of clot time is subjective, requires training and can vary between operators and institutions^17^, existing tests use optical and magnetic Hall-effect sensors to automatically track changes in plasma viscosity and turbidity^18^. Mechanical approaches use specialized hardware to analyze the movements of steel balls^19^, iron fillings^20^, and magnets^21^ in the plasma sample. Other automated approaches include electrochemical sensors that measure capacitance and resistance^22–25^, quartz crystal resonators^26^, micro-resonators^18^, and centrifugal-type microfluidic platforms^27^. Though laboratory clotting assays enable high-throughput testing, they may not be able to provide rapid turnaround for anticoagulant therapy in emergency room and intensive care units, which often require results within 30 minutes and around-the-clock availability^28^. Commercial point-of-care systems eliminate these delays but can be expensive for field use, in resource-constrained settings and for in-home patients, who cite cost of PT/INR home devices as the primary barrier to self-testing^13^.

Unlike these conventional coagulation tests, our design is low-cost, with a total material cost of around $0.03, and requires only a lightweight (17 g), compact (70 × 27.5 × 60.9 mm), 3D-printed plastic smartphone attachment, disposable plastic cup, and tiny copper particle. Further, our solution leverages on-board vibration motors and cameras that are ubiquitous on modern smartphones and are accessible in resource-constrained environments. Finally, ours is an automated solution that does not require manual observation or interpretation of clotting data.

**Fig. 1:**
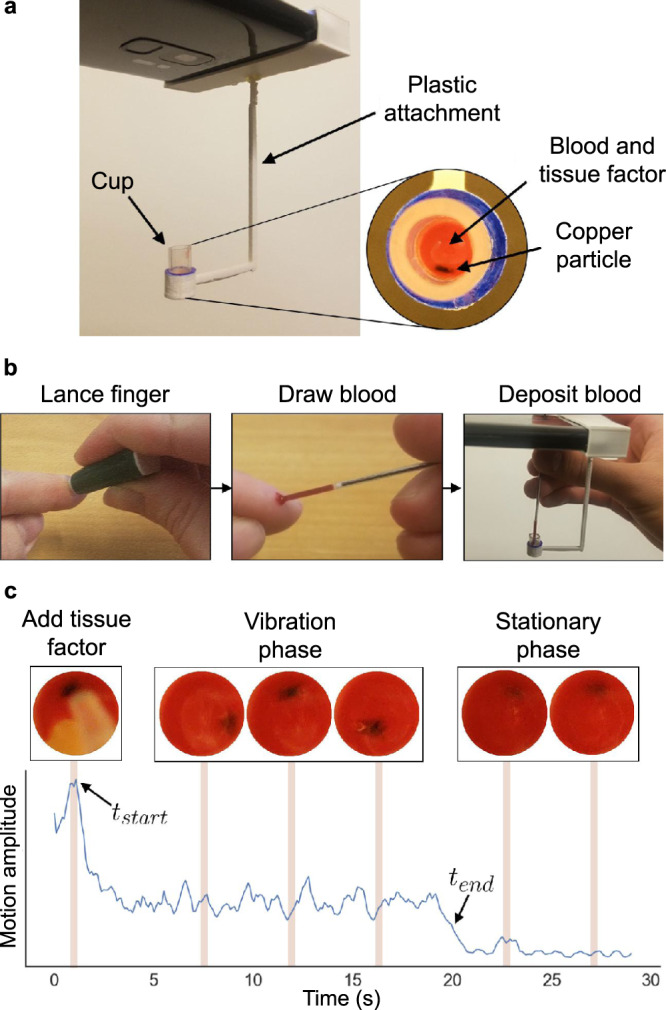
Blood coagulation testing using a smartphone. **a** Schematic of the system: a plastic attachment containing a cup with 10 μl of whole blood, 20 μl of tissue factor, and a copper particle. The smartphone’s vibration motor is coupled to the attachment and vibrates the particle, which is captured with the camera. **b** A workflow (from left to right) showing how whole blood is added to the cup in our system. **c** The phone captures the motion of the particle starting from when the tissue factor is added to the blood (*t*_start_). The particle moves freely in the blood when it is in a liquid state. When the blood coagulates, the particle’s motion reduces (*t*_end_).

Our system leverages the smartphone’s onboard vibration motor to vibrate a small silicone cup (Fig. 1). The smartphone is coupled to the cup via a custom 3D-printed plastic attachment. The ‘L’ shaped structure of the plastic attachment is constructed using thin material to allow the smartphone’s vibrations to propagate to the cup holder while reducing dampening. In addition, the cup holder makes physical contact with both the bottom and sides of the cup to ensure maximal physical energy transfer from the attachment to the cup. The plastic attachment is designed to position the cup under the smartphone’s camera and its width is determined by the smartphone’s dimensions.

The cup holds 10–20 μl of plasma or whole blood and a small copper particle, provided as part of our design (Fig. 1). A thromboplastin activator is added to this mixture to activate the extrinsic pathways of the coagulation cascade. The copper particle is a 1 mm long AWG22 copper wire coated with dark blue ink for increased visibility. It is lightweight and moves freely in response to the phone’s vibration. In addition, it is non-porous, which prevents it from soaking up any reagent. We chose copper material because it is also low-cost, widely available, and easily cut to short lengths. The form factor of our proof-of-concept design could be further optimized for patient use by integrating the copper particle into single-use disposable cups that are coated with a dried form of thromboplastin^29,30^.

**Fig. 2:**
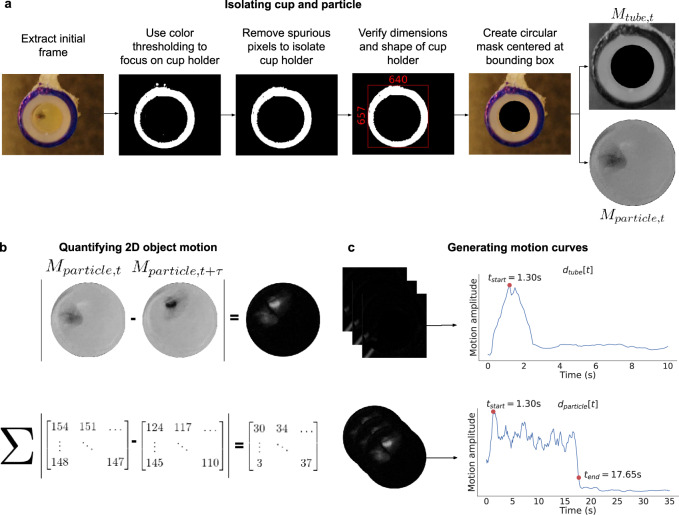
Workflow to compute PT from the smartphone video. **a** Color thresholding and image processing techniques are applied to identify the cup holder and to create a mask that captures the motion of the capillary tube or pipette, *M*_tube,*t*_. We then isolate the interior of the cup containing the particle, *M*_particle,*t*_. **b** The L1 norm is applied between masked images to quantify the amount of 2D motion between video frames. **c** The most prominent peak in the motion curve *d*_tube_[*t*] marks the start of the PT measurement, *t*_start_. The knee of the particle’s motion curve *d*_particle_[*t*] denotes the particle’s transition from motion to stasis, and marks the end of the PT measurement, *t*_end_.

When the blood or plasma sample is not coagulated, the smartphone’s vibrations cause the copper particle to move and rotate within the sample. As the sample coagulates, its increasing viscosity constricts the particle’s movements, slowing its projected 2D motion as seen by the camera (see Supplementary Movie 1). The particle’s movement is recorded optically by the smartphone camera and analyzed to calculate PT/INR. In particular, our algorithm identifies when the activator is added and computes the coagulation time automatically. We isolate the 2D movement of the particle from the background and perform a correlation analysis of video frames to generate motion curves (Fig. 2). The start of a measurement is detected when the activator is dispensed to the cup. A steep drop in the particle’s motion curve indicates the particle’s stationary state and marks the mixture’s clot time. These motion curves are then analyzed to algorithmically identify the start and end time points of the measurement and compute the PT/INR values (see the “Methods” section). Internal checks are performed before, during, and after a measurement to ensure the test has been performed properly (Supplementary Table 2). If all internal checks have passed, the PT/INR result is displayed.

As our system only processes video frames captured every 100 ms to calculate PT/INR, any smartphone camera that can capture video at 10 fps or greater is able to record the clotting process. All modern smartphones capable of capturing video records at a minimum of 24 or 30 fps, which is the standard frame rate for video recordings. We note also that when the video resolution was downsampled to 960 × 540 there was no change in computed PT/INR values for plasma and blood samples, which shows that lower resolution smartphone cameras can capture the clotting process. The most common resolution for video recording on modern smartphones is 1920 × 1080.

### Clinical testing on plasma samples

We evaluated our system using 140 anonymized plasma samples from the University of Washington Medical Center (UWMC). A subset of these samples included plasma from patients who were undergoing treatment at the UWMC Anticoagulation clinic and were thus likely to have high PT/INR values. Samples were marked with PT/INR values obtained from a clinical-grade coagulation analyzer (Diagnostica Stago STA R Max). They were collected and tested on our smartphone setup within 12 h of being drawn from the patients as prior studies have shown no significant change in PT values for samples up to 24 h from blood draw^31,32^. The plasma samples were stored at room temperature prior to measurement on our system. The PT/INR values of the plasma samples ranged from 11.4–49.8 s and 0.9–5.3, respectively, with a mean value of 20.1 s and 1.8 and a median value of 17 s and 1.4 (Supplementary Fig. 2a).

Since the smartphone measurements were performed hours after the plasma samples were collected, the samples (and the thromboplastin activator) were heated in a water bath at 37 °C (approximating the body temperature) for three minutes. The measurement was then conducted at room temperature and humidity. As the test takes less than a minute, ambient temperature and humidity within normal limits do not significantly affect the performance of the system (see Supplementary Table 1 and Supplementary Materials for details). Twenty microliters of the plasma was added into the cup with the copper particle and placed into the smartphone attachment. The smartphone’s vibration motor was turned on to vibrate continuously, and the camera began recording. Forty microliters of the activator was then added into the cup. Samples were tested on a Samsung Galaxy S9 phone and each test was performed twice to evaluate test–retest performance.

**Fig. 3:**
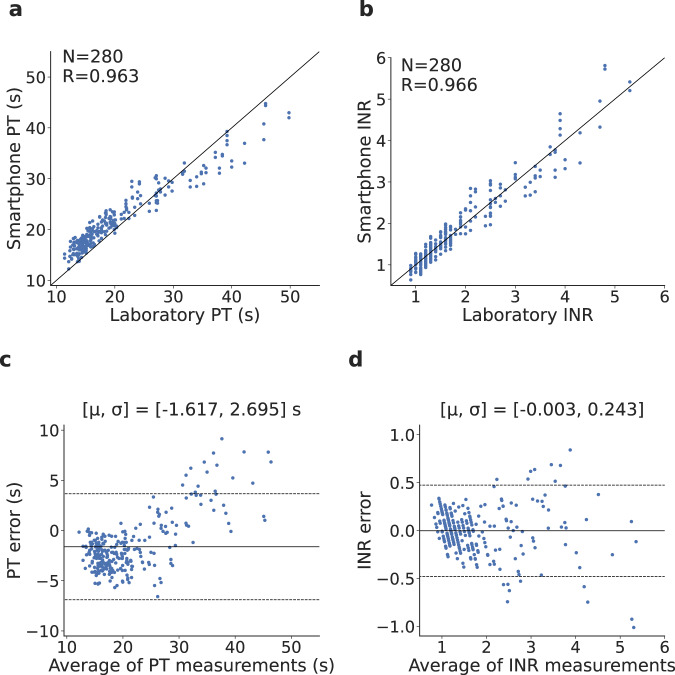
Plasma clinical testing results. **a**–**d** Correlation and Bland-Altman plots comparing plasma PT/INR values from the smartphone system and the clinical-grade coagulation analyzer. In the Bland-Altman plot, μ is the mean error and σ is the standard deviation (SD) of the errors, the solid line represents the mean error and the dotted lines represent the 95% limits of agreement. Source data are provided as a Source Data file.

The PT/INR values computed by the smartphone system were compared against the laboratory PT/INR values. The inter-class correlation coefficient for PT/INR was *R* = 0.963 and *R* = 0.966, respectively (Fig. 3a, b). This is within the accuracy range of 0.77–0.97 for commercial point-of-care testing coagulometers^33^. Bland-Altman analysis demonstrated a bias error of 1.617 s for PT, with 16 of 280 measurements samples falling outside the 95% agreement limits (Fig. 3c). Similar analysis for INR showed a bias error of −0.003, with 17 of 280 samples falling outside the 95% agreement limits (Fig. 3d). We note that increased variance at higher INRs may be due to variability in the international sensitivity index (ISI) of the tissue factor used by the laboratory versus our smartphone^34^. To evaluate test–retest reliability, each plasma sample was tested twice. The intra-assay coefficient of variation (CV) between duplicate measurements of 140 plasma samples was 3.62% for PT and 6.14% for INR, which is within the range of 1.4–8.4% found using commercial point-of-care testing coagulometers^33^.

For 100 of these plasma samples, we also conducted a manual tilt-tube test in parallel with the smartphone system. For these tests, 50 μl of plasma and 100 μl of activator were used; larger amount of plasma enabled more consistent testing with manual readings. The container was tilted back and forth until a clot formed. Clot times were noted by eye and recorded using a stop watch. Head-to-head testing demonstrated a PT/INR correlation between the manual test and the ground truth of *R* = 0.960 for both PT/INR, which is similar to the correlation obtained by our smartphone system (Supplementary Fig. 1a, b). The Bland-Altman analysis for PT showed a bias of −1.140 s, with five of 100 measurements falling outside the 95% agreement limits (Supplementary Fig. 1c). Similar analysis for INR showed a bias of 0.098, with six of 100 measurements falling outside these limits (Supplementary Fig. 1d).

### Coagulopathy testing

We also evaluated our system on an additional 79 plasma samples collected from two sites, from patients with a known coagulopathy. Specifically, we obtained samples across a broad range of coagulopathic causes including patients who had disseminated intravascular coagulation (DIC) (*n* = 8), liver disease (*n* = 19), trauma (*n* = 8), or conditions requiring anticoagulation such as extracorporeal membrane oxygenation (ECMO) (*n* = 6) or who were on heparin (*n* = 17) or warfarin (*n* = 13) to treat a medical condition (Table 1). The samples were obtained from both UWMC and Harborview Medical Center (HMC), and included samples from trauma patients in the emergency department undergoing blood transfusion. The PT/INR values of the plasma samples ranged from 12.6 to 67.2 s and 1–7.6, respectively, with a mean value of 22.2 s and 2.0 and a median value of 19.2 s and 1.6 (Supplementary Fig. 2b). We note that the mean PT/INR for these patients is double that of a normal INR of 1.0. Samples were collected and tested using the same procedure as the first evaluation on plasma samples.

**Table 1:** Coagulopathies of patients.

Coagulopathy *n* (%)	Normal INR (≤1.1)	Elevated INR (>1.1)
Disseminated intravascular coagulation	0 (0.0)	8 (13.1)
Heparin therapy	14 (77.8)	3 (4.9)
Liver disease	0 (0.0)	19 (31.1)
Trauma	0 (0.0)	8 (13.1)
Acute injury	0 (0.0)	3 (4.9)
Perioperative	0 (0.0)	5 (8.2)
Warfarin therapy	0 (0.0)	13 (21.3)
Atrial fibrillation	0 (0.0)	7 (11.5)
Congenital heart disease	0 (0.0)	1 (1.6)
Deep vein thrombosis	0 (0.0)	2 (3.3)
Valve disease	0 (0.0)	3 (4.9)
Other	4 (22.2)	10 (16.4)
Leukemia/Lymphoma	1 (5.6)	1 (1.6)
Extracorporeal membrane oxygenation	3 (16.7)	3 (4.9)
Familial hypercoagulability	0 (0.0)	1 (1.6)
Multiple anticoagulants	0 (0.0)	4 (6.6)
Other	0 (0.0)	1 (1.6)

**Fig. 4:**
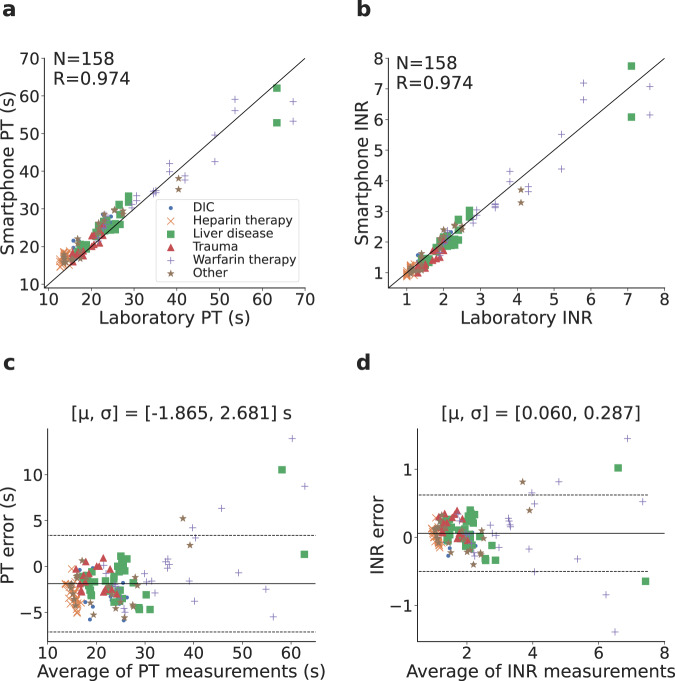
Coagulopathy testing results. **a**–**d** Correlation and Bland-Altman plots comparing coagulopathic plasma PT/INR values from the smartphone system and the clinical-grade coagulation analyzer. In the Bland-Altman plot, μ is the mean error and σ is the standard deviation (SD) of the errors, the solid line represents the mean error and the dotted lines represent the 95% limits of agreement. Source data are provided as a Source Data file.

The inter-class correlation coefficient between the smartphone system and the clinical-grade coagulation analyzer was *R* = 0.974 for both PT/INR. For samples with an elevated INR > 1.2, the correlation coefficient within each of the coagulopathy categories ranged from 0.890 to 0.977. Bland-Altman analysis demonstrated a bias error of −1.865 s for PT, with six of 158 measurements samples falling outside the 95% agreement limits (Fig. 4c). Similar analysis for INR showed a bias error of 0.060, with nine of 158 samples falling outside the 95% agreement limits (Fig. 4d).

On subgroup analysis, patients who were on heparin therapy did not demonstrate a substantially elevated PT/INR with a mean of 13.9 s and 1.1 respectively. Heparin affects coagulation of the intrinsic pathway, while PT/INR assesses the extrinsic pathway so this is an expected negative control result. Warfarin does directly affect the extrinsic pathway and this group had the highest average PT/INR of the different conditions studied with a mean of 36.2 s and 3.6 respectively. Individuals with an INR > 4.5 are at a nearly six fold increased risk of a bleeding event^35^. Of the four patients with an INR > 4.5, our system had an INR error of 14% compared to laboratory measurements. We included patients who quickly developed coagulopathy after a traumatic event as well as those with longstanding liver disease or on anticoagulation therapy. In all tested cases, the PT/INR assessment tools were well correlated, indicating that this device can be used with a variety of coagulopathies.

### Clinical testing on whole blood samples

We also evaluated the performance of our smartphone system on 80 anonymized samples of whole blood (blue top, 3 mL collected in sodium citrate) tested against the results of a commercial point-of-care PT/INR test meter (Coag-Sense, CoaguSense Inc.). In order to test the device capabilities across a range of coagulopathic conditions and PT/INR values, samples associated with particular diagnosis or from a particular clinic were preferentially obtained (the anticoagulation clinic and emergency department at Harboview Medical Center) with elevated PT/INR on laboratory testing, no other information about the sample than the patient coagulopathy was obtained. 30 of 80 samples were collected and tested on our smartphone system within 4 hours of being drawn from patients; twenty-two of the samples were tested within 4–12 h of blood draw; the remaining samples were refrigerated and tested more than 12 h later. The whole blood samples were collected under the same conditions as the plasma ones. PT/INR from the commercial test meter ranged from 13.5 to 39.0 s and 1.1–3.6, respectively, with a mean of 21.7 s and 1.9 and median of 21.1 s and 1.8 (Supplementary Fig. 2c).

The same amount of whole blood (10 μl) and thromboplastin activator (20 μl) were used for testing with the smartphone system and the commercial PT/INR meter. With the commercial meter, the whole blood and activator were each added to the test strip in quick succession as soon as the measurement started. The same thromboplastin activator was used for the commercial PT/INR meter and the smartphone system. As before, since each whole blood sample exceeded one milliliter, we tested each twice with the smartphone to evaluate test–retest performance.

**Fig. 5:**
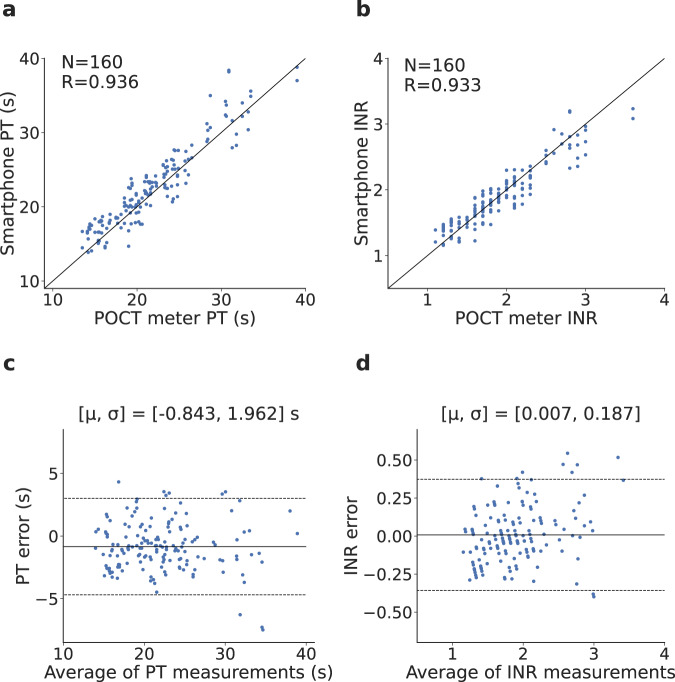
Whole blood clinical testing results. **a**–**d** Correlation and Bland-Altman plots comparing whole blood PT/INR values from the smartphone system and the commercial POCT coagulometer. In the Bland-Altman plot, μ is the mean error and σ is the standard deviation (SD) of the errors, the solid line represents the mean error and the dotted lines represent the 95% limits of agreement. Source data are provided as a Source Data file.

The inter-class correlation coefficient was computed between the smartphone system and the commercial PT/INR meter (Fig. 5a, b). Across the 160 measurements of PT/INR, correlation coefficients were *R* = 0.936 and *R* = 0.933, respectively. These are within the accuracy range obtained by commercial point-of-care testing coagulometers^33^. Bland-Altman analysis for PT showed a bias of −0.843 s, with nine of 160 measurements falling outside the 95% limits (Fig. 5c). The bias for INR was 0.007, with ten of 160 measurements falling outside these limits (Fig. 5d).

We also evaluated the test–retest performance for whole blood testing. The intra-assay CV between the duplicate measurements was 5.39% for both PT/INR, which again is within the precision range obtained from commercial point-of-care testing coagulometers^33^ (Supplementary Table 2).

We evaluated the consistency of plasma and whole blood testing in our system by measuring a low and high PT/INR sample ten times in a row (Table 2). The low PT/INR (14.3 s and 1.1) plasma sample had a CV of 6.62% and 11.39%, respectively, while the high PT/INR (27.3 s and 2.5) sample had a CV of 4.52% and 7.76%, respectively. The low PT/INR (15.4 s and 1.3) whole blood sample had a CV of 8.06% for both PT/INR, while the high PT/INR (33.2 s and 3.0) sample had a CV of 6.64% for both PT/INR.

**Table 2:** Precision testing of plasma and whole blood samples for low and high PT/INR values.

	Low PT/INR plasma sample	High PT/INR plasma sample	Low PT/INR whole blood sample	High PT/INR whole blood sample
Ground truth PT (s)	14.3	27.3	15.4	33.2
Ground truth INR	1.1	2.5	1.3	3.0
Smartphone PT (s)	16.6 ± 1.1	25.9 ± 1.2	15.3 ± 1.2	33.5 ± 2.2
Smartphone INR	1.1 ± 0.1	2.3 ± 0.2	1.3 ± 0.1	2.8 ± 0.2

The table shows the mean for each benchmark and an SD computed across ten measurements. Source data are provided as a Source Data file.

### Benchmark testing

Finally, we present benchmark testing across several design conditions. Testing was performed on plasma samples that were still able to clot more than 12 h after collection. Each scenario was tested three times on a single plasma sample, and the mean particle motion curve was plotted, with a shaded region representing one standard deviation from the mean. We omit the high amplitude spike at the start of the motion curve, which captures the activator addition into the solution, to more clearly identify the amplitude differences towards the end of the measurement. The motion curves are smoothed and cropped to show the area around the knee of the curve for visualization purposes.

**Fig. 6:**
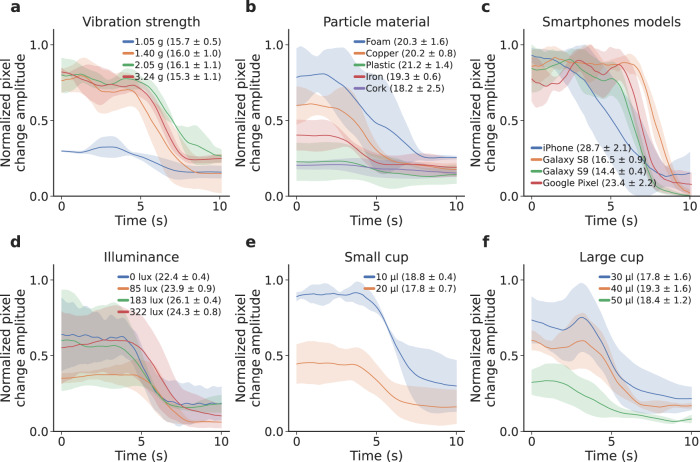
Benchmark testing across different scenarios. The system was evaluated across (**a**) different vibration strengths, **b** different particle materials, **c** different smartphone models, **d** illuminance levels, and **e**, **f** different volumes of plasma in a small and large cup. The figure shows the mean and SD computed across three measurements. Source data are provided as a Source Data file.

We first considered the effect of vibration strength on particle motion (Fig. 6a). We varied vibration strength across a range of amplitudes from the Samsung Galaxy S9 smartphone’s built-in vibration motor and measured vibration strength at the cup holder with an accelerometer (Bosch Sensortec BMA400). For different vibration strengths, we computed the Euclidean norm from the *x*, *y* and *z* axes of the accelerometer and averaged them over a period of five seconds. The copper particle did not move as much at vibrations of 1.05 g compared to higher vibration values; however, for the full range of tested vibration values, the particle moved freely enough that the algorithm could detect when it stopped moving. Although the magnitude of particle movement did not change significantly for vibration strengths above 2.05 g in this benchmark, a vibration strength of 3.24 g was selected for our main evaluations: more viscous plasma samples required a higher vibration strength for the particle to move freely. Though the phone can produce vibrations of up to 3.77 g, we found that at those vibration levels, the particle could escape from the top of the container and small droplets of plasma spilled during measurement.

Second, we evaluated how different particle materials affected system performance (Fig. 6b). We found that small particles of copper, iron and foam could vibrate freely and thus were detectable. The copper and iron particles sank below the plasma surface and rattled around at the bottom of the container. The foam particle floated on the surface of the plasma. Although foam and iron particles produced sufficient motion in the fluid, we did not select them for the main evaluations since they were harder to reuse for subsequent samples. The plastic and cork particles both sank to the bottom of the container but still exhibited a small amount of motion that was detectable by the camera and algorithm. However, the amplitude differences between the vibratory and stationary states were small enough to be challenging to track across a larger number of measurements and samples.

Third, we evaluated how our system works with other smartphone models (Fig. 6c). The Samsung Galaxy S8 had a similar geometry to the Samsung Galaxy S9 used in the main evaluations, and produced comparable motion curves when the attachment was coupled to the top of the phone. The iPhone’s vibration motor had a lower strength, and the plastic attachment was placed in more direct contact with the right hand side of the phone where vibration amplitude was highest. While the Google Pixel’s vibration motor was located on the bottom of the phone, the motor was strong enough to produce comparable motion curves.

Fourth, we examined how ambient illumination affected the system (Fig. 6d). When the ambient incident light level was 0 lux with no external light sources, the smartphone’s flash was used to illuminate the cup and particle. For higher levels, the flash was turned off. At 0 lux, the flash reflected off the plasma and continued to move even after the plasma has coagulated. This results in high frequency motion seen throughout the motion curve. However, the particle’s transition to a stationary state could still be readily identified. At other illuminance levels, the camera’s ISO was increased and shutter speed decreased so the particle would be visible (see Materials and methods). The motion curves, and PT/INR at these illuminance levels were comparable and the particle’s stopping points could be identified.

Fifth, we examined the effects of different plasma volumes from 10 to 50 μl (Fig. 6e, f). In each case, the amount of thromboplastin added to the mixture was increased proportionately. We note that 10 μl represents the amount of whole blood (approximately one drop) required for use by the commercial point-of-care PT/INR meter, and 50 μl represents the amount of plasma used by the clinical-grade coagulation analyzer. Volumes of 10 and 20 μl were tested in the 4 mm diameter plastic tube containers used in our main evaluations. Larger volumes of plasma were tested in a larger 8 mm diameter plastic cup to accommodate the increased volume of fluid. The particle’s movement was more subtle at these higher volumes, but this can be attributed to the choice of container, which may have dampened vibrations. Across all volumes, measures of PT/INR were comparable, and the particle’s motion and transition to a stationary state was detectable by the algorithm.

Sixth, we evaluate the effect of placing the smartphone system on a variety of different surface materials (Supplementary Fig. 3). We chose both soft materials including foam and cloth, as well as hard materials that could form a tabletop surface including wood, metal and glass. We note that while the bottom half of the smartphone rests on the surface, the top half containing the vibration motor and attachment hangs over the edge of the surface to accommodate the shape of the attachment. We find that across all tested materials, the motion curves were similar and there was no significant change in PT/INR values. This shows that the smartphone vibration motor is strong enough to cause the particle to vibrate even when it is placed on common soft materials.

Seventh, we measured the effect of temperature and humidity on PT/INR for a single plasma sample on our smartphone system in a laboratory incubator (IVYX Scientific 5L Incubator). The incubator was used to control the ambient temperature around the smartphone and attachment to temperature values within the range of 18–32 °C). This range was selected to match the normal temperature conditions of use for the Coag-Sense POCT meter^36^. The relative humidity of the incubator ranged from 44–59% in this evaluation as measured by a hygrometer (Thermometer World). Across this temperature and humidity range, average PT/INR stayed relatively constant ranging from 12.9 to 13.6 s and 0.7–0.8 respectively (Supplementary Table 1). We note that this range is within the standard deviation of precision testing of a single low PT/INR plasma sample which is 1.1 s and 0.1 respectively (Table 2). As PT/INR testing typically takes less than a minute, ambient temperature and humidity, within normal limits, do not have a significant effect on system performance.

Finally, we examined the effect of diluting a whole blood sample with normal saline on PT/INR measurements from our smartphone system and a point-of-care PT/INR test meter. The hemoglobin levels of each dilution were also measured using a commercial hemoglobin meter (Mission Plus Hemoglobin Meter, Acon Laboratories Inc.). Dilution levels were selected such that the hemoglobin levels of the dilutions covered the range of 8.5–18.7 g/dL, which is the range within which PT/INR testing is to be performed in patients, as recommended by our institution^37^. We note that these dilution levels also affect the opacity of the blood sample. Supplementary Fig. 4 shows the dilution range from 0 to 50%, which covers hemoglobin concentrations in the range of 5.4–18.6 g/dL. As the dilution level increases from 0% to 50%, PT/INR values from our system and the POCT meter increase. PT/INR increased from 19.6 s (1.7) to 29.3 s (2.6) for the POCT meter and 18.5 s (1.5) to 32.2 s (2.7) for the smartphone system. This is because increased dilution levels dilute the coagulation factors that are activated as part of a PT assay, resulting in a longer time for a clot to form.

## Discussion

Our smartphone-based micro-mechanical clot detection system demonstrates strong correlation with laboratory and point of care PT/INR tests for both plasma and whole blood. 279 of 280 (99.6%) plasma measurements and 100 of 100 whole blood measurements fall within the allowable differences for INR testing, greater than the 90% threshold set by the International Organization of Standardization for this type of device^38^.

A key advantage of leveraging smartphone hardware for medical purposes is that custom electronics and hardware do not have to be designed, which lowers the development costs typically required to obtain regulatory approval. Specifically, under the FDA’s Mobile Medical Applications (MMA)^39,40^ and Software as a Medical Device (SaMD)^41–43^ guidance, the agency does not regulate the smartphone hardware, and only regulates custom software functions. The FDA has cleared or approved commercially available MMAs that use sensors like microphones and cameras to perform medical diagnostics^44^.

Our work is not without limitations. While our clot detection system was effective for testing both plasma and whole blood, limitations of existing plasma and whole blood tests still apply. Laboratory testing methods typically rely on plasma testing, which eliminates the effects of hematocrit concentration on the result^45,46^. However, plasma testing requires a centrifuge to separate plasma from red blood cells; while commercial centrifuges can cost as little as $10–20, cheaper hand-powered ones could also be used for this purpose^47^. Still, studies demonstrate variability across tests performed on different machines and with different reagents^48–50^.

Point-of-care PT/INR testing is performed using capillary blood, which can be obtained by the patient or caregiver. We evaluated our system using whole blood venous samples; there are known differences between capillary and whole blood but prior comparisons of point-of-care PT/INR devices have shown small but unlikely clinically significant differences^51^. Obtaining capillary blood is simpler, but can be susceptible to unreliable results in certain conditions. Thus, current commercial whole-blood based devices are not recommended for use in patients with anemia or polycythemia^52^. Further, as capillary blood samples are often drawn by less trained individuals, such as a patient or caregiver, it is important to follow proper procedures to avoid contamination, which could impact coagulation^53^. In addition, point-of-care testing INR values that exceed 3.5 do not correlate as well with laboratory tests compared with lower INR values and require reassessment in a laboratory testing environment^54^. For higher INR values, these devices are most useful in alerting the patient and provider to the need for further medical attention given the higher risk of bleeding. We note also that fibrinogen deficiency and its effects on PT/INR levels were not evaluated on our device, further studies are required to evaluate these effects.

The patient population tested using our smartphone device is different from the expected end user. As our samples were anonymous, except for the cause of coagulopathy in some cases, it was not possible for us to discern if the tested individuals would be eligible for point of care PT/INR monitoring. However, as our inclusion criteria were far more broad than typical hospital or provider criteria for home monitoring, we anticipate this modality to work as well if not better in this subgroup.

Due to the optical nature of our detection system, the opacity of the mixture affects the visibility of the particle’s movement. The evaluation with whole blood was conducted using 10 μl of blood, which left the copper particle visible to the camera. Unlike plasma, which is translucent, whole blood is opaque, and a smaller amount is needed to avoid visually obscuring the movement of the copper particle. Similarly, using larger quantities of plasma in a small diameter container will obscure the movement of the particle. Further, in our clinical studies, the plasma, whole blood and thrombplastin activator were heated to body temperature in a commercial water bath. We could, in the future, prototype a low-cost heating element design in the form of a Peltier tile ($0.62)^55^, a temperature sensor ($0.19)^56^, a transistor ($0.07)^57^, and a microcontroller ($0.99)^58^ to regulate temperature output and be powered via a USB-C connection present on many modern smartphones.

The technique described here incorporates the most commonly used laboratory reagents for clot initiation, which do require refrigeration at 2–8 °C, easily obtained by a standard home refrigerator and have a seven day shelf life once reconstituted. This could be sufficient for a rural clinic that serves multiple patients on blood-thinning medications. Alternatively, other formulations have been developed which could be painted on to the bottom of the cup and are stable for months at room temperature and are sufficient for home use^29,30^.

Looking forward, while the design of the current system is optimized to detect PT, future system design could be adapted to perform related coagulation tests—such as partial thrombplastin time (PTT) or thrombin time (TT)—which can be performed on commercial coagulation analyzers using mechanical and optical techniques. Other coagulation tests that rely on viscoelastic measurements, such as thromboelastography or rotational thromboelastometry, could potentially leverage our principle of operation to track clot changes over longer time scales. Further work and studies are required to empirically validate the full potential of our design for such applications.

Point-of-care PT/INR testing can improve time in therapeutic range for anticoagulation users and improve quality of life, but it is currently inaccessible to millions of patients^6^. Smartphone-based micro-mechanical clot detection utilizes small blood volumes easily obtained by the patient or caregiver through a finger stick. Given the pervasiveness of smartphones, our rugged proof-of-concept system has the potential to reduce prior barriers to entry due to its low cost and may increase use in low-resource environments. Future studies are required to assess the performance of our system in these real-world environments.

## Methods

Our evaluations were approved by the University of Washington Institutional Review Board. Anonymized and de-identified plasma and whole blood samples were collected from the University of Washington Medical Center and its Anticoagulation clinic and Harborview Medical Center. PT/INR measurements on the smartphone system were performed by a student with no prior experience in liquid lab work after a few minutes of supervised training. Informed consent was obtained from patients prior to sample collection, with no compensation.

### Video processing algorithm

Our algorithm has three main steps. First, it identifies which pixels in a given frame correspond to the cup holder. Doing this lets us determine which pixels correspond to the interior of the cup where the particle vibrates and which pixels correspond to the cup and cup holder, where the pipette or capillary tube entering and exiting the frame can be seen. Second, it identifies the start of the PT measurement, *t*_start_, by tracking the motion of the tube as it enters and exits the frame. Third, the algorithm computes the end of the PT measurement, *t*_end_, by tracking the particle inside the cup. We now describe each step in more detail.

#### Identifying the cup holder pixels

The algorithm’s first step is to isolate the pixels corresponding to the cup from other pixels within a given video frame *F*_*t*_ = [*R*_*t*_, *G*_*t*_, *B*_*t*_]. To more easily locate the cup, the cup holder is marked with permanent blue ink. Our algorithm then applies an RGB color filter to identify the pixels corresponding to the blue ink. This produces pixels corresponding to the cup holder as well as other spurious pixels in the frame that may be in the same color range.

To isolate the pixels corresponding to the cup holder from spurious ones, we use the flood-fill algorithm to find the list of connected components in the frame. This algorithm defines a connected component as regions where all pixels in the region are connected along the horizontal, vertical or diagonal axis. The largest connected component is then selected as the most likely candidate region for the cup holder. Figure 2 shows these connected components, where, due to random noise, this algorithm returned multiple connected components representing not just the cup holder but also many spurious pixel regions. The list of connected components is then filtered to a single connected component representing the cup holder. To do this, we verify that the bounding box, *bbox*, around the candidate object is more than 500 × 500 pixels in size to ensure the connected components filter has not returned any small pixel regions that correspond to spurious noise. Further, to ensure that the pixel region is circular in shape, we check that the difference between the width and height of the bounding box is no greater than 200 pixels.

#### Identifying *t*_start_

This algorithmic step identifies the time at which the PT measurement begins. This corresponds to the instance when the pipette or capillary tube is used to add activator to the blood or plasma sample. To capture the tube motion and separate it from the particle’s motion, we track all pixels within the cup holder defined by *bbox* but not pixels within the cup containing the vibrating particle, as shown in Fig. 2.

To do this, we first reduce the amount of information channels required for processing. Our algorithm converts each RGB video frame with color channels *R*, *G*, *B* at timepoint *t* to a frame with grayscale intensity values. We then create a circular mask *mask* that captures the interior of the cup and particle. This mask is placed at the center of the bounding box *bbox*. The diameter of this mask is half the bounding box’s length or width, whichever is greater. This means that the frame includes pixels that represent the cup and cup holder, and only the interior of the cup with the particle is masked out. The frame is then cropped to the location of bounding box *bbox* to produce what we call the *tube frame*, *M*_tube,*t*_, where *M*_tube,*t*,*i*,*j*_ represents the pixel at location (*i*, *j*).

1 $${M}_{{{{{\rm{tube}}}}},t,i,j}=\left\{\begin{array}{ll}0\hfill \quad &(i,j)\in mask\\ {F}_{t,i,j}\quad &{{{{{{\rm{otherwise}}}}}}}\,\end{array}\right.$$

The n ext step is to quantify the motion of the capillary tube or pipette between two masked frames (*M*_tube,*t*_, *M*_tube,*t*+*τ*_). Doing so lets us determine when the tube has entered the frame, which is used to calculate *t*_start_. The motion between two masked frames is quantified by taking the L1-norm (the sum of the absolute difference between pixel intensities) between frames of dimension *W* × *H*, to generate a motion curve, *d*_*tube*_[*t*]:

2 $${d}_{{{{{\rm{tube}}}}}}[t]=\mathop{\sum }\limits_{i=0}^{W}\mathop{\sum }\limits_{j=0}^{H}| {M}_{{{{{\rm{tube}}}}},t,i,j}-{M}_{{{{{\rm{tube}}}}},t+\tau ,i,j}| ,t\in [0,6,\ldots ,60]$$

The video is captured at 60 frames per second, and we compute this value between frames at every *τ* interval every six frames, i.e., *τ* = 6. This lets us capture motion at a resolution of 100 ms. The motion curve is computed for the first ten seconds of the video within which the activator is expected to be added. *t*_start_ is then calculated using this motion curve, *d*_tube_[*t*]. Specifically, the algorithm applies a moving average filter with a window size of 10 to *d*_tube_[*t*] and crops the tube motion curve of any trailing motion artifacts that occur after the tube enters and exits the frame. This is done by finding the knee of the curve. At a high level, the knee (or elbow) of a curve is defined as the point where the curve transitions from a high to a low slope region. In this case, the high slope region refers to the motion of the pipette or capillary tube, and the low slope one refers to the subsequent period of no motion or small motion artifacts, which we want to crop out.

The knee point of a motion curve *d*[*t*_0…*N*_] is defined as the point *p* where the combined root mean square error (RMSE) of a linear regression on segments *d*[*t*_0…*p*−1_] and *d*[*t*_*p*+1…*N*_] is jointly minimized. Linear regression on *d*[*t*_0…*p*−1_] and *d*[*t*_*p*+1…*N*_] yield the coefficient vector (slope, intercept), ${\overrightarrow{\beta }}_{0,p-1}$ and ${\overrightarrow{\beta }}_{p+1,N}$, respectively. The coefficients are computed using least squares estimation:

3 $${\overrightarrow{\beta }}_{m,n}={({X}_{m,n}^{T}{X}_{m,n})}^{-1}{X}_{m,n}^{T}d[{t}_{m\cdots n}]$$

Here, *X*_*m*,*n*_ is a Vandermode matrix with the following entries:

4 $${X}_{m,n}=\left[\begin{array}{cc}1&m\\ 1&m+1\\ \vdots &\vdots \\ 1&n\end{array}\right]$$

The knee point is then computed by minimizing the error function *J*(*p*, *d*[*t*]) below:

5 $$\mathop{\min }\limits_{p}J(p,d[t])=	\; \sqrt{\sum {\left(d[{t}_{0\ldots p-1}]-{X}_{0,p-1}\cdot {\overrightarrow{\beta }}_{0,p-1}\right)}^{2}}\\ 	+ \sqrt{\sum {\left(d[{t}_{p+1\ldots N}]-{X}_{p+1,N}\cdot {\overrightarrow{\beta }}_{p+1,N}\right)}^{2}}$$

We minimize Eq. (5) by iterating through all points, *p*, to obtain the knee point. Once the motion curve, *d*_tube_[*t*], has been cropped to the knee point, the peak identifying the entry of the tube into the frame can be identified. The peak with maximum prominence in this cropped range represents the point of maximum motion change between frames and is marked as *t*_start_. We note that in our studies, we use a pipette but since the algorithm uses motion rather than identifying the pipette itself, it also works well with a capillary tube.

#### Identifying *t*_end_

The final algorithmic step identifies the end of the PT measurement, *t*_end_. We compute *t*_end_ as the point when the particle in the cup transitions from a moving state to a stationary state. To isolate the movements of vibration within the cup from the rest of the frame, we first crop the frame *F*_*t*_ to within the bounding box around mask. The mask is then applied so only pixels within the circular region of the cup are visible. This produces *M*_particle,*t*_, where *M*_particle,*t*,*i*,*j*_ represents the pixel at location (*i*, *j*):

6 $${M}_{{{{{\rm{particle}}}}},t,i,j}=\left\{\begin{array}{ll}0\hfill\quad &(i,j)\,\notin\, mask\\ {F}_{t,i,j}\quad &\,{{\mbox{otherwise}}}\,\end{array}\right.$$

To quantify the motion of the particle vibrating within the cup, we calculate a motion curve *d*_particle_[*t*] for the particle. This curve quantifies motion between a pair of masked frames (*M*_particle,*t*_, *M*_particle,*t*+*τ*_) for the full length of the video (*t* ∈ [0, *N*]), where *N* is the total number of frames in the video:

7 $${d}_{{{{{\rm{particle}}}}}}[t]=\mathop{\sum }\limits_{i=0}^{W}\mathop{\sum }\limits_{j=0}^{H}| {M}_{{{{{\rm{particle}}}}},t,i,j}-{M}_{{{{{\rm{particle}}}}},t+\tau ,i,j}| ,t\in [0,N]$$

Next, we calculate *t*_end_ the end time of the PT measurement from the motion curve *d*_particle_[*t*]. The particle’s motion curve is smoothed with a moving average filter with window size of ten. The algorithm only analyzes the particle’s motion 10 s seconds after the start of measurement: 10 s corresponds to the lowest INR value of 0.8 that can be detected on the commercial POCT coagulometer used for testing, and it represents a lower bound on PT values. We note that the lowest PT value across all plasma and whole blood testing was 11.4 s.

The period of time after the particle has stopped moving is then removed from analysis. To do this, we normalize the particle motion curve in the range of [0, 1]. We then crop the trailing portion of the curve after the curve amplitude drops below the 0.01 mark. The knee point of the cropped motion curve of *d*_particle_[*t*] corresponds to the point where the particle transitions from motion to a stationary state and is marked as *t*_end_, the end of the PT measurement.

### PT/INR computation

The PT value is computed by computing the time it takes for the particle to stop moving in the blood or plasma sample, *PT* = *t*_end_ − *t*_start_. INR levels are used since it normalizes the PT values across different labs and different test methods and allows for easier comparison. INR is traditionally computed as

8 $${{{{{\rm{INR}}}}}}={\left(\frac{{{{{\rm{P}}}}}{{{{{\rm{T}}}}}}_{test}}{{{{{\rm{P}}}}}{{{{{\rm{T}}}}}}_{normal}}\right)}^{{{{{\rm{ISI}}}}}+\alpha }$$

Here, PT_*normal*_ is the PT value corresponding to plasma samples in the normal PR range; ISI is the international sensitivity index, which compares the batch of tissue factor used with an international reference tissue factor; and *α* is a correction factor.

For plasma testing, a value of 16 s for PT_*normal*_ was calculated as the average PT measurements from our system across the 33 plasma samples within the normal INR range of 0.9–1.1. The ISI for the lot of thromboplastin used was 1.23. A correction factor *α* of 0.48 was used to normalize the calculated INR values for our system. For the coagulopathy evaluation, PT_*normal*_ was set to 16 s similar to the first evaluation on plasma. The ISI for the thrombplastin lot was 1.31, and a correction factor of 0.2 was used. For whole blood testing, PT_*normal*_ was set to 12 s, which corresponds to an INR value of 1.0 on the commercial PT/INR test meter. The ISI for the thromboplastin lot used in these experiments was 1.31, and a correction factor *α* of −0.31 was used.

### Smartphone application

We designed a custom Android application on a Samsung Galaxy S9 to perform measurements. The vibration motor on the Samsung Galaxy S9 has a resonant frequency of 159 Hz. The motor was set to vibrate continuously while the camera recorded the clotting process. The camera had an ISO of 320, 1/60 shutter speed, 5500 K white balance and captured frames at the maximum frame rate. For the benchmark experiments, an iOS app was also developed to vibrate the plastic attachment. The Samsung Galaxy S8, iPhone 5s and Google Pixel 3a phones used for benchmark experiments had a resonant frequency of 159, 231 and 153 Hz respectively. A vibration strength of 200 units was selected for the Samsung Galaxy S8 and S9 as this was high enough to move the particle freely without causing it to escape from the top of the container. The maximum vibration strength of 255 units was selected for the Google Pixel. The maximum vibration strength was also selected for the iPhone. The resonant frequency of the phone’s vibration motor is fixed and cannot be controlled in software. The video recordings were captured at 30 fps with the default settings in the camera app of each phone. For benchmark experiments with different illuminance levels, ISO100 and a shutter speed of 1/1000 was used at 0 lux, while ISO800 and 1/30 shutter speed was used at other illuminance levels. 30 fps was used for testing different illuminance levels due to the high shutter speed selected.

### Attachment design

Smartphone vibration motors have been used to extract the surface tension on a liquid (e.g., urine) by analyzing the wave pattern^59,60^. This however requires orders of magnitude more surface area to capture multiple waves and hence much larger quantities of liquid. Our smartphone attachment with the copper particle addresses this problem and works with 10–20 μl of blood or plasma. The smartphone attachment for the Samsung Galaxy S9 consists of a 3D-printed plastic (PLA) frame that couples to the front of the smartphone, a 60 mm long vertical stem and a container holder. The length of the vertical stem was selected so that the container and particle were within the smartphone camera’s focal length. The container holder has a diameter of 6 mm to accommodate the diameter of the container. The container was a silicone tube with an inner diameter of 4 mm, an outer diameter of 6 mm, and a height of 10 mm. Hot glue was used to create a water-tight seal around the end of the silicone tube. The Samsung Galaxy S8 has a similar geometry as the Galaxy S9, and the attachment used for the S9 fit on the S8. For the iPhone 5s and Google Pixel 3a, the ends of the S9 attachment were cut off so the optimal placement of the attachment could be found. For the iPhone 5s, the attachment was secured in contact in the top right corner of the phone. For the Google Pixel 3a, the attachment was secured in contact at the top of the phone. The cup holder was also larger with an inner diameter of 11.5 mm, to accommodate the larger plastic cup with an outer diameter of 11 mm. Both attachments were secured using rubber bands for the experiments. The length of the vertical stem was the same for the attachment used across these phones.

### Cost analysis

The thrombplastin activator was STA Neoplastine CI + 10 (Diagnostica Stago), which cost $52.16 for 120 ml of solvent and lyophilized thromboplastin. At this price, the 20 μl of activator needed for our system would cost 0.87 cents^61^. The plastic smartphone attachment is made of 2.10 m of PLA filament and can be printed, with a material cost of $0.0021^62^. The silicone cup costs $0.03, with a unit cost of $4.80 for 2 m of tubing^63^. A single copper particle costs <0.03 cents^64^.

### Statistical analysis

Inter-class class correlation was calculated using Pearson’s correlation coefficient for correlation plots. The bias error (mean error) and 95% limits of agreement (LOA) were computed for the Bland-Altman plots. LOA was computed as 1.96 times the standard deviation of the error. For precision testing, CV was computed as the standard deviation divided by the mean of samples. For duplicate testing, intra-assay CV was computed as in ref. ^65^ where individual CVs were computed for each duplicate sample, and the mean CV across all samples was reported as the overall intra-assay CV. For benchmark testing, where repeated testing was performed under the same experimental conditions, PT/INR results were reported as mean ± standard deviation. Statistical analysis was performed using MATLAB, and the figures were generated using the Python matplotlib and seaborn library.

### Reporting summary

Further information on research design is available in the Nature Research Reporting Summary linked to this article.

## Supplementary information

Supplementary information

Description of Additional Supplementary Files

Supplementary Video 1

Reporting Summary

## Data Availability

All data supporting the findings from this study are available within the article and its supplementary information. Source data are provided with this paper.

## Source data

Source Data

## Code availability

Code is available at https://github.com/uw-x/blood_coagulation.
