# Supplementary material for: Micro-mechanical blood clot testing using smartphones
Source: Nat Commun. 2022 Feb 11;13:831. doi: 10.1038/s41467-022-28499-y (PMC8837659; doi:10.1038/s41467-022-28499-y)
Supplement: Supplementary file 1 — Supplementary information [file 41467_2022_28499_MOESM1_ESM.pdf]

Micro-mechanical blood clot testing using smartphones

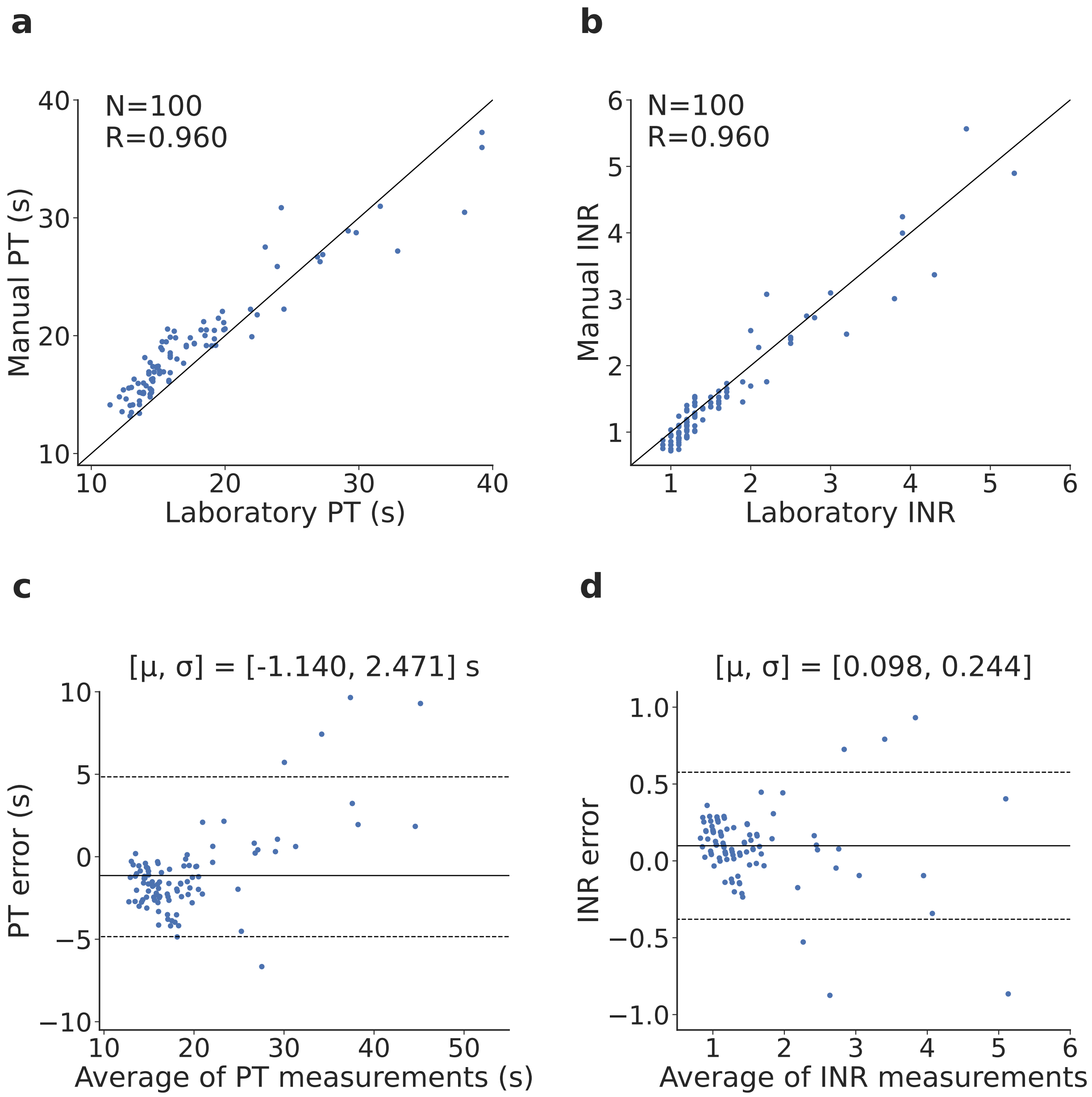

Supplementary Figure 1: **Manual tilt-tube testing result. a-d** Correlation and Bland-Altman plots comparing PT/INR values obtained from a manual tilt-tube test with the clinical-grade coagulation analyzer. In the Bland-Altman plot, μ is the mean error and σ is the standard deviation (SD) of the errors, the solid line represents the mean error and the dotted lines represent the 95% limits of agreement. Source data are provided as a Source Data file.

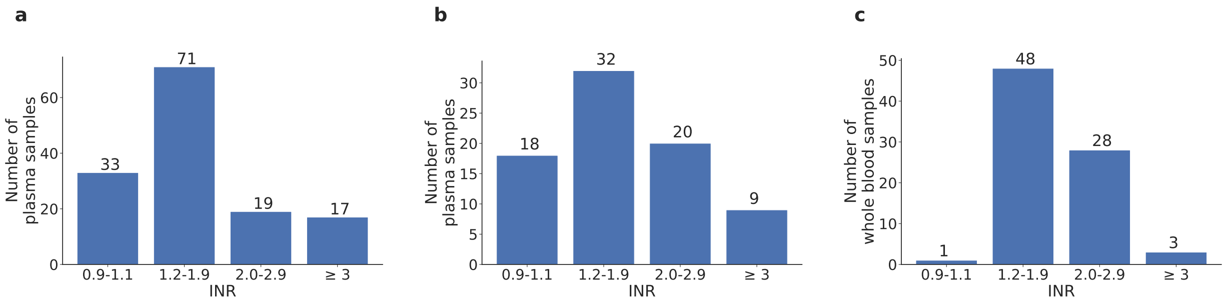

Supplementary Figure 2: **Distribution of reference standard INRs.** **a** Histogram of INRs from clinical-grade coagulation analyzer across 140 plasma samples. **b** Histogram of INRs from clinical-grade coagulation analyzer across 79 plasma samples for the coagulopathy evaluation. **c** Histogram of INR values from commercial POCT coagulometer across 80 whole blood samples.

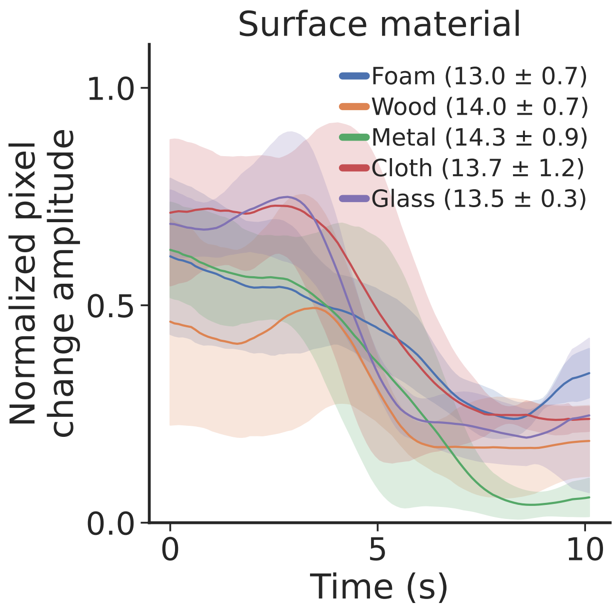

Supplementary Figure 3: **Effect of surface material on system performance.** The figure shows the mean and SD computed across three measurements. Source data are provided as a Source Data file.

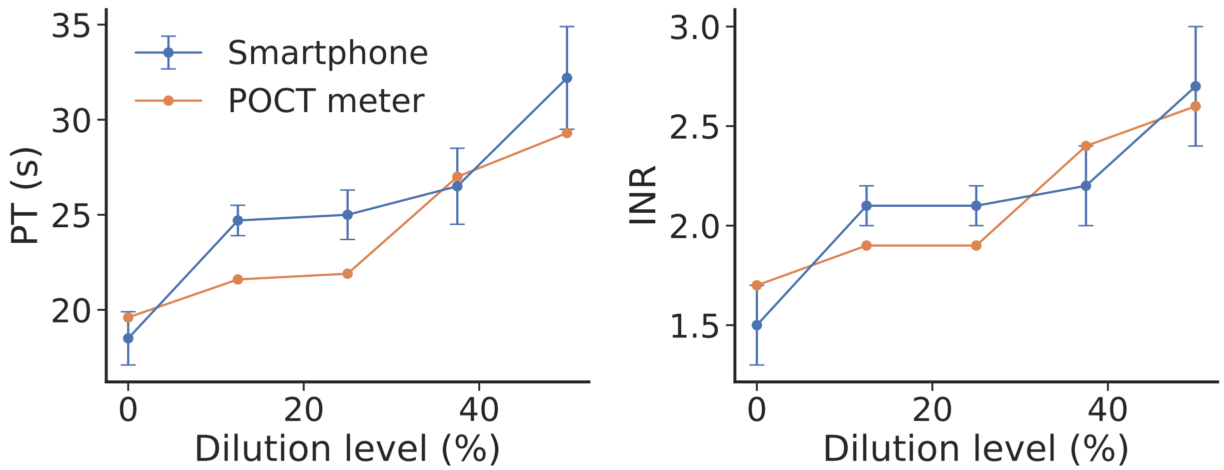

Dilution level (%)	Hemoglobin (g/dL)
0	18.6
12.5	15.4
25	14.1
37.5	7.5
50	5.4

Supplementary Figure 4: **Effect of hemodilution on system performance.** PT/INR is measured for whole blood diluted with saline water at different dilution levels on our smartphone system and a commercial POCT PT/INR meter. The figure shows the mean across three measurements and the errors bars show an SD. The table shows the hemoglobin measured using a commercial hemoglobin meter. Source data are provided as a Source Data file.

Temperature (°C)	Relative humidity (%)	PT (s)	INR
18	59	13.6 ± 0.9	0.8± 0.1
21	57	13.2 ± 0.3	0.7± 0.0
24	54	13.5 ± 0.8	0.8± 0.1
27	50	12.9 ± 0.8	0.7± 0.1
30	47	13.2 ± 0.8	0.7± 0.1
32	44	13.0 ± 0.8	0.7± 0.1

Supplementary Table 1: **Effect of temperature and relative humidity on system performance.** PT/INR is measured three times for a single plasma sample on our smartphone-based system in a laboratory incubator set to different temperatures. Commercial POCT PT/INR meters like Coag-Sense (*1*) have an operating temperature range of 18 – 32°C. The table shows the mean and standard deviation across three measurements. Source data are provided as a Source Data file.

Timepoint	Internal check	Trigger condition
Pre-measurement	Cup holder not detected	Color marking cup holder not detectable with color thresholding.
Pre-measurement	Cup holder improperly positioned	Cup holder is not in the center of the frame. Occurs if the attachment is not fit snugly with the phone.
Pre-measurement	Cup not detected	Color marking the bottom of the cup is not detectable with color thresholding, suggesting cup is not present.
Pre-measurement	Sample not detected	Color thresholding only shows an empty cup in the frame.
During measurement	Particle not moving or particle not in cup	Video frames are too similar, indicating minimal motion.
During measurement	Abrupt motion change	High amplitude change between video frames could indicate particle has flown out of cup, or user has removed the cup prior to the end of the measurement.
Post-measurement	Clot time too short	Clotting time was marked as less than 8 seconds.
Post-measurement	Clot time too long	Clotting time was marked as more than 90 seconds.

Supplementary Table 2: **Internal checks and trigger conditions.** Listing of internal checks performed before, during, and after a given measurement, which are used to approve a result.

Device name	Correlation coefficient	Coefficient of variation
CoaguChek (*2*)	0.877	4.90 – 8.00
CoaguChek XS (*3, 4*)	0.810 – 0.980	1.40 – 5.92
CoaguChek XS Plus (*5*)	0.974	2.00
CoaguChek Vantus (*6*)	0.910	3.30
CoaguSense (*7*)	0.920	2.53
Immedia Prothrombin Time System (*8*)	0.920	3.50
HemoChron Junior (*2*)	0.834	7.00 – 13.90
Rapidpoint Coag (*2*)	0.923	14.40 – 22.30
Smartphone-based system (our work)	0.933	5.39

Supplementary Table 3: **Comparison against commercial PT/INR monitors.** The table compares published performance measures of commercial PT/INR monitors and our smartphone-based test for INR of whole blood samples tested by trained operators against a laboratory reference standard. System precision is shown for duplicate INR measurements of whole blood samples.

Supplementary references

1. Coag-Sense Professional User's Manual.
2. Nowatzke, W. L. *et al.* Whole blood international normalization ratio measurements in children using near-patient monitors. *Journal of pediatric hematology/oncology* **25**, 33–37 (2003).
3. 510(k) Decision Summary (CoaguChek XS system) URL https://www.accessdata.fda.gov/cdrh_docs/pdf6/K060978.pdf.
4. Christensen, T. D. & Larsen, T. B. Precision and accuracy of point-of-care testing coagulometers used for self-testing and self-management of oral anticoagulation therapy. *Journal of Thrombosis and Haemostasis* **10**, 251–260 (2012).
5. 510(k) Decision Summary (CoaguChek XS Plus system) URL https://www.accessdata.fda.gov/cdrh_docs/pdf7/K071041.pdf.
6. 510(k) Decision Summary (CoaguChek Vantus System and CoaguChek XS PT Test Strips) URL https://www.accessdata.fda.gov/cdrh_docs/reviews/K170960.pdf.
7. 510(k) Decision Summary (CoaguSense Self-Test Prothrombin Time/INR Monitoring System) URL https://www.accessdata.fda.gov/cdrh_docs/reviews/K093243.pdf.
8. 510(k) Decision Summary (Immedia Prothrombin Time System) URL https://www.accessdata.fda.gov/cdrh_docs/reviews/K050243.pdf.
