# Supplementary material for: Micro-mechanical blood clot testing using smartphones
Source: Nat Commun. 2022 Feb 11;13:831. doi: 10.1038/s41467-022-28499-y (PMC8837659; doi:10.1038/s41467-022-28499-y)
Supplement: Supplementary file 2 — Description of Additional Supplementary Files [file 41467_2022_28499_MOESM2_ESM.docx]

**Description of Additional Supplementary Files**

**File Name:** Supplementary Video 1

**Description:** Video illustrating workflow for tracking coagulation of whole blood on a smartphone.
