# Supplementary material for: Micro-mechanical blood clot testing using smartphones
Source: Nat Commun. 2022 Feb 11;13:831. doi: 10.1038/s41467-022-28499-y (PMC8837659; doi:10.1038/s41467-022-28499-y)
Supplement: Supplementary file 4 — Reporting Summary [file 41467_2022_28499_MOESM4_ESM.pdf]

nature portfolio

Corresponding author(s): Justin Chan, Kelly Michaelsen, Shyamnath Gollakota

Last updated by author(s): Dec 18, 2021

Reporting Summary

Nature Portfolio wishes to improve the reproducibility of the work that we publish. This form provides structure for consistency and transparency in reporting. For further information on Nature Portfolio policies, see our Editorial Policies and the Editorial Policy Checklist.

Statistics

For all statistical analyses, confirm that the following items are present in the figure legend, table legend, main text, or Methods section.

n/a	Confirmed	
☐	☒	The exact sample size (n) for each experimental group/condition, given as a discrete number and unit of measurement
☐	☒	A statement on whether measurements were taken from distinct samples or whether the same sample was measured repeatedly
☒	☐	The statistical test(s) used AND whether they are one- or two-sided<br>*Only common tests should be described solely by name; describe more complex techniques in the Methods section.*
☒	☐	A description of all covariates tested
☒	☐	A description of any assumptions or corrections, such as tests of normality and adjustment for multiple comparisons
☐	☒	A full description of the statistical parameters including central tendency (e.g. means) or other basic estimates (e.g. regression coefficient) AND variation (e.g. standard deviation) or associated estimates of uncertainty (e.g. confidence intervals)
☒	☐	For null hypothesis testing, the test statistic (e.g. F, t, r) with confidence intervals, effect sizes, degrees of freedom and P value noted<br>*Give P values as exact values whenever suitable.*
☒	☐	For Bayesian analysis, information on the choice of priors and Markov chain Monte Carlo settings
☒	☐	For hierarchical and complex designs, identification of the appropriate level for tests and full reporting of outcomes
☐	☒	Estimates of effect sizes (e.g. Cohen's d, Pearson's r), indicating how they were calculated

Our web collection on statistics for biologists contains articles on many of the points above.

Software and code

Policy information about availability of computer code

Data collection: The default camera app for was used for recording videos. A custom app was used for vibrating the phone.

Data analysis: Custom python and MATLAB scripts were used to calculate PT and INR information from a recorded video. The following software was used:
Matlab R2021a
opencv-python 4.5.2
matplotlib 3.4.2
seaborn 0.11.1

Custom analysis code:
https://github.com/uw-x/blood_coagulation

For manuscripts utilizing custom algorithms or software that are central to the research but not yet described in published literature, software must be made available to editors and reviewers. We strongly encourage code deposition in a community repository (e.g. GitHub). See the Nature Portfolio guidelines for submitting code & software for further information.

Data

Policy information about availability of data

All manuscripts must include a data availability statement. This statement should provide the following information, where applicable:

- Accession codes, unique identifiers, or web links for publicly available datasets
- A description of any restrictions on data availability
- For clinical datasets or third party data, please ensure that the statement adheres to our policy

Source data are provided with this paper. All data generated in this study are available as part of the Source data.

Field-specific reporting

Please select the one below that is the best fit for your research. If you are not sure, read the appropriate sections before making your selection.

[x] Life sciences [ ] Behavioural & social sciences [ ] Ecological, evolutionary & environmental sciences

For a reference copy of the document with all sections, see nature.com/documents/nr-reporting-summary-flat.pdf

Life sciences study design

All studies must disclose on these points even when the disclosure is negative.

Sample size	Sample sizes were chosen based on plasma and whole blood sample availability, and whether a sufficiently representative number of samples were obtained for a range of normal and abnormal INR values.
Data exclusions	No data was excluded.
Replication	The findings can be replicated.<br>We performed repeated testing of samples on the smartphone for plasma, coagulopathic, and whole blood samples for the clinical studies. Each sample was measured twice.<br>For benchmark testing testing, each experimental condition was measured three times.
Randomization	Randomization of samples is not relevant. Samples were not allocated into different groups.
Blinding	Investigators were not blinded. Blinding would have made it difficult to identify samples that span a sufficiently wide range of PT/INR values and coagulopathies for this study.

Reporting for specific materials, systems and methods

We require information from authors about some types of materials, experimental systems and methods used in many studies. Here, indicate whether each material, system or method listed is relevant to your study. If you are not sure if a list item applies to your research, read the appropriate section before selecting a response.

Materials & experimental systems

n/a	Involved in the study	
[x]	[ ]	Antibodies
[x]	[ ]	Eukaryotic cell lines
[x]	[ ]	Palaeontology and archaeology
[x]	[ ]	Animals and other organisms
[ ]	[x]	Human research participants
[x]	[ ]	Clinical data
[x]	[ ]	Dual use research of concern

Methods

n/a	Involved in the study	
[x]	[ ]	ChIP-seq
[x]	[ ]	Flow cytometry
[x]	[ ]	MRI-based neuroimaging

Human research participants

Policy information about studies involving human research participants

Population characteristics	Anonymized plasma and whole blood samples were collected from the University of Washington Medical Center (UWMC) and Harborview Medical Center (HMC). A subset of these samples included plasma from patients who were undergoing treatment at the UWMC Anticoagulation clinic.
Recruitment	Informed consent was obtained from patients prior to sample collection. The inclusion criteria for our cohort were patients that were undergoing treatment at the University of Washington Medical Center (UWMC) and Harborview Medical Center (HMC) and obtaining PT/INR testing. Since we compute PT/INR we cannot anticipate any manner in which our inclusion criteria would bias our primary results.
Ethics oversight	The University of Washington Institutional Review Board (IRB) authorized the research.

Note that full information on the approval of the study protocol must also be provided in the manuscript.
